# Supplementary material for: Identifying Key Predictors of Smoking Cessation Success: Text-Based Feature Selection Using a Large Language Model
Source: medRxiv. 2025 Jun 20:2025.06.18.25329854. Preprint. [Version 1] doi: 10.1101/2025.06.18.25329854 (PMC12204296; doi:10.1101/2025.06.18.25329854)
Supplement: Supplement 1 [file media-1.pdf]

Appendix to "Identifying Key Predictors of Smoking Cessation Success: Text-Based Feature Selection Using a Large Language Model"

Thuy T. T. Le[1*], Jiongxuan Yang[2], Zimo Zhao[3], Kaidi Zhang[3], Wenjun Li[4], and Yan Hu[3]

[1]University of Michigan School of Public Health, Department of Health Management and Policy, Ann Arbor, MI, USA. [*]Corresponding email: thuyttle@umich.edu

[2]University of Michigan School of Public Health, Department of Biostatistics, Ann Arbor, MI, USA

[3]The Chinese University of Hong Kong, School of Data Science, Shenzhen, China

[4]University of Massachusetts Lowell, Department of Public Health and Center for Health Statistics, Lowell, MA, USA

Prompt template:

Below is the prompt that we used in this study

Prompt = { "*As a researcher specializing in tobacco cessation behavior analysis, after thoroughly reviewing the literature, your task is to score the importance of the variable,* ***variable_desciption****, in predicting whether an individual will abstain from smoking during the 12-month period preceding their interview, which is scheduled to take place two years from now. Provide a numeric score between 1 and 100 to reflect the variable's importance, with 1 being the least important and 100 being the most important. Ensure your response is formatted precisely as: 'Score: XX', where XX represents your numeric score. Following the score, include a concise reasoning of your rating starting with 'Reasoning:', and limited to one or two sentences.*"}

where ***variable_desciption*** is the description of each variable from Table A1.

Table A1: The parameter space of the optimized XGBoost hyperparameters.

**Hyperparameters**	eta	max_depth	min_child_weight	subsample	colsample_bytree
**Parameter space**	[0.01, 0.1]	[4L, 25L]	[0, 25]	[0.5, 1]	[0.5, 1]

Table A2: The exhaustive list of the top 45 variables together with their descriptions and their means and standard deviations of important scores provided by GPT-4.1.

**Category**	**Variable name**	**Variable description**	**Mean score (SD)**	**Mean SHAP value**
Smoking Frequency and Habits	R05R_A_NUMDAYS_CIGS	Adult Past 30 Day Cigarette Smoking Frequency	90 (3)	0.16220
Nicotine Dependence	R05_AC9022	Consider yourself a smoker	85 (2)	0.05796
Smoking Frequency and Habits	R05R_A_MINFIRST_CIGS	Adult Number of Minutes from Waking Up to Smoking First Cigarette	85 (1)	0.05690
Social and Environmental Influences	R05_AX0071	People who are important to you: Their views on using tobacco	75 (3)	0.04514
Social and Environmental Influences	R05_AN0255	Level of agreement: Most people I spend time with are tobacco users (current established, recent former established or current experimental non-electronic tobacco users)	81 (2)	0.02817
Nicotine Dependence	R05_AN0070	Level of agreement: Smoking/using tobacco product(s) really helps me feel better if feeling down	78 (1)	0.02796
Other Tobacco Product Usage Patterns	R05R_A_EDY_EPRODS	Adult Current Every Day Electronic Nicotine Product User	79 (3)	0.02557
Health Harm Perceptions and Concerns	R05_AX0105	Extent to which you are worried that using/your past use of tobacco products will damage your health in the future	77 (2)	0.02498
Nicotine Dependence	R05_AN0060	Level of agreement: Usually want to smoke/use tobacco product(s) right after waking up	90 (2)	0.02472
Other Tobacco Product Usage Patterns	R05R_A_EDY_TOB	Adult Every Day Tobacco User	95 (1)	0.02469
Enjoying Sensation	R05_AC9045	Level of Agreement: Enjoy sensation in throat and chest when smoking	75 (3)	0.02439
Cessation Efforts and Intentions	R05_AN0105	In the past 12 months have you tried to quit smoking/using tobacco product(s)	76 (4)	0.02389

Social and Environmental Influences	R05_AR1045	Statement that best describes rules about smoking a combustible tobacco product inside your home	76 (3)	0.02009
Nicotine Dependence	R05_AN0030	Level of agreement: Urges keep getting stronger if don't/Still have urges to smoke/use tobacco product(s)	85 (1)	0.01889
Cessation Efforts and Intentions	R05_AN0235	Plans to quit smoking/using tobacco product(s) for good	76 (3)	0.01829
Social and Environmental Influences	R05_AX0741_10	People who are important to you use the following products: None of the above	78 (3)	0.01730
Nicotine Dependence	R05_AN0100	Level of agreement: After not smoking/using tobacco product(s) for a while, need to smoke/use tobacco product(s) in order to keep self from experiencing any discomfort	87 (1)	0.01693
Smoking Frequency and Habits	R05_AC9053	Smoke cigarettes more frequently during the first hours after waking compared to rest of the day	85 (1)	0.01559
Nicotine Dependence	R05_AN0055	Level of agreement: Finds self reaching for tobacco product(s) without thinking about it	79 (2)	0.01509
Nicotine Dependence	R05_AN0045	Level of agreement: My tobacco product(s) smoking/use is out of control/My urge to smoke/use tobacco product(s) is out of control	83 (2)	0.01479
Nicotine Dependence	R05_AN0065	Level of agreement: Can only go a couple of hours without smoking/using tobacco product(s)	86 (1)	0.01478
Nicotine Dependence	R05_AN0035	Level of agreement: Tobacco product(s) control me	79 (2)	0.01457
Nicotine Dependence	R05_AN0010	Consider yourself to be addicted to tobacco product(s)	76 (3)	0.01429
Nicotine Dependence	R05_AN0095	Level of agreement: After not smoking/using tobacco product(s) for a while, need to smoke/use tobacco product(s) in order to feel less restless and irritable	86 (2)	0.01320
Nicotine Dependence	R05_AN0085	Level of agreement: Would find it really hard to stop smoking/using tobacco product(s)	84 (1)	0.01258
Nicotine Dependence	R05_AN0050	Level of agreement: Frequently smoke/use tobacco product(s) without thinking about it	83 (2)	0.01168
Other Tobacco Product Usage Patterns	R05R_A_SDY_TOB	Adult Some Day Tobacco User	81 (3)	0.01110
Nicotine Dependence	R05_AN0025	Level of agreement: Frequently crave tobacco product(s)	85 (1)	0.01098
Nicotine Dependence	R05_AN0015	Has strong cravings to smoke/use tobacco product(s)	85 (1)	0.01084
Health Harm Perceptions and Concerns	R05_AX0104	Extent to which using/past use of tobacco products damaged your health	77 (3)	0.01044
Nicotine Dependence	R05_AN0090	Level of agreement: Would find it hard to stop smoking/using tobacco product(s) for a week	76 (3)	0.01014
Nicotine Dependence	R05_AC9054	Smoke cigarettes even if ill in bed all day	85 (2)	0.00839

Health Harm Perceptions and Concerns	R05_AN0110	Do you believe that smoking/using tobacco products is causing/caused a health problem or made it worse	82 (3)	0.00723
Nicotine Dependence	R05_AN0020	Felt like you really needed to smoke/use tobacco product(s)	85 (1)	0.00634
Other Tobacco Product Usage Patterns	R05R_A_EDY_CIGAR	Adult Current Every Day Cigar Smoker	85 (2)	0.00575
Other Tobacco Product Usage Patterns	R05R_A_P30D_GRILLO	Adult Past 30 Day Cigarillo Smoker	77 (4)	0.00481
Other Tobacco Product Usage Patterns	R05R_A_CUR_EXPR_TOB	Adult Current Experimental Tobacco User	85 (1)	0.00355
Other Tobacco Product Usage Patterns	R05R_A_P12M_GTRAD	Adult Past 12 Month Traditional Cigar Smokers	79 (4)	0.00350
Other Tobacco Product Usage Patterns	R05R_A_CUR_EXPR_EPRODS	Adult Current Experimental Electronic Nicotine Product User	78 (2)	0.00157
Other Tobacco Product Usage Patterns	R05R_A_P30D_GTRAD	Adult Past 30 Day Traditional Cigar Smoker	77 (4)	0.00130
Other Tobacco Product Usage Patterns	R05R_A_CUR_EDSD_GFILTR	Adult Current Every Day/Some Day (Without Threshold) Filtered Cigar Smoker	75 (4)	0.00127
Other Tobacco Product Usage Patterns	R05R_A_SDY_EPRODS	Adult Current Some Day Electronic Nicotine Product User	77 (3)	0.00124
Other Tobacco Product Usage Patterns	R05R_A_CUR_EDSD_HOOK	Adult Current Every Day/Some Day (Without Threshold) Hookah Smoker	76 (3)	0.00110
Other Tobacco Product Usage Patterns	R05R_A_CUR_EDSD_GTRAD	Adult Current Every Day/Some Day (Without Threshold) Traditional Cigar Smoker	77 (3)	0.00080
Other Tobacco Product Usage Patterns	R05R_A_CUR_ESTD_GTRAD	Adult Current Established Traditional Cigar Smoker	78 (4)	0.00009
